# Long‐term low dose nitisinone therapy in adults with alkaptonuria shows no cognitive decline or increased severity of depression

**DOI:** 10.1002/jmd2.12272

**Published:** 2022-03-17

**Authors:** Andrew S. Davison, Gin Hughes, Joanne A. Harrold, Pam Clarke, Rebecca Griffin, Lakshminarayan R. Ranganath

**Affiliations:** ^1^ Department of Clinical Biochemistry and Metabolic Medicine, Liverpool Clinical Laboratories Royal Liverpool University Hospital Liverpool UK; ^2^ Department of Psychology University of Liverpool Liverpool UK; ^3^ Liverpool Cancer Trials Unit University of Liverpool Liverpool UK

**Keywords:** Alkaptonuria, BDI‐II, cognitive functioning, depression, nitisinone, WAIS‐IV

## Abstract

Little is documented on whether nitisinone‐induced hypertyrosinaemia alters cognitive functioning or leads to worsening depression in alkaptonuria (AKU). Wechsler Adult Intelligence Scale‐IV (WAIS‐IV) and Beck Depression Inventory‐II (BDI‐II) assessments were performed before and annually following treatment with nitisinone 2 mg daily to assess the impact on cognitive functioning and severity of depression. Serum tyrosine concentrations were also measured annually. *WAIS‐IV:* 63 patients (27 females/36 males: mean age[years] [±standard deviation, range] 55.7[13.7, 26–79]; 60.3[9.6, 19–75]) were included at baseline for assessment of: verbal comprehension (VC), perceptual reasoning (PR), working memory (WM), and processing speed (PS) using separate indices. Over the 6‐year period studied 43, 39, 36, 29, 26 and 15 patients had annual assessments. Using a longitudinal model (age and sex adjusted) no significant differences were observed in any of the indices over this period, apart from VC which showed a significant increase after adjustment for sex (*p* < 0.05). *BDI‐II:* 74 patients (32 females/42 males: mean age[years] [±standard deviation, range] 56.1[13.2, 26–79]; 42 males, 51.5[16.3, 19–70]) were included at baseline. Over the 7‐year period studied 48, 47, 38, 34, 32, 24 and 12 patients had annual assessments. No significant differences in BDI‐II scores were observed when compared to baseline. Hypertyrosinaemia was observed in all patients following treatment with nitisinone (*p* < 0.001, at all annual visits). Serum tyrosine was not correlated with WAIS‐IV sub‐test indices or BDI‐II scores pre‐ or post‐nitisinone therapy. These findings suggest that treatment with nitisinone does not affect cognitive functioning and or lead to increased severity of depression.

**Synopsis**

Nitisinone does not affect cognitive functioning and or lead to increased severity of depression in patients with AKU.

## 1 INTRODUCTION

Alkaptonuria (AKU, OMIM: 203500) is a rare inherited metabolic disorder of the tyrosine metabolic pathway occurring in 1 in 250 000 in the general population^1^ (see Davison et al.^2^ for a recent review). Central to the treatment of AKU is the lowering of the increased circulating concentration of homogentisic acid (HGA), which is pathognomonic of the disease. This is achieved through the use of nitisinone, a reversible competitive inhibitor of hydroxyphenylpyruvate dioxygenase (EC 1.13.11.27). The metabolic complication of this therapy is the well‐known hypertyrosinaemia, which has been widely reported in AKU^1^, ^3^, ^4^, ^5^, ^6^, ^7^, ^8^, ^9^ and hereditary tyrosinaemia type 1 (HT‐1, OMIM: 276700).^10^, ^11^, ^12^, ^13^

Much of the literature available on hypertyrosinaemia in AKU relates to the clinical consequences that have been observed, such as corneal keratopathy and skin rash.^4^, ^6^, ^14^, ^15^, ^16^ These particular side effects typically resolve when nitisinone is stopped or dosage is modified. It has been postulated in HT‐1 that hypertyrosinaemia may be linked to the neurocognitive deficits observed, through altered neurotransmitter metabolism such as lower intelligence quotient; motor abilities, impaired executive functioning, and social cognition; and schooling problems.^17^, ^18^, ^19^, ^20^, ^21^ A more recent study however^22^ demonstrated no significant correlation between tyrosine and intelligence quotient at school age. In principle, alterations in neurotransmitter metabolism may also lead to changes in mood, although this has not been reported in HT‐1 or AKU.

Several mechanisms have been proposed for altered neurotransmitter metabolism including: (i) increased transport of tyrosine into the brain and a consequent decrease in the transport of other large neutral aromatic amino acids via the large neutral amino acid transporter 1 into the brain (i.e., tryptophan), (ii) increased dopamine and decreased serotonin in the central nervous system and (iii) altered serotonin metabolism due to the direct inhibition of tryptophan hydroxylase (EC 1.14.16.4) activity by tyrosine.^23^, ^24^, ^25^, ^26^

To date there are no reports of altered cognitive functioning or central neurotransmitter metabolism in AKU patients with hypertyrosinaemia. In order to assess central neurotransmitter metabolism either cerebrospinal fluid or a brain biopsy are required as they directly reflect neurotransmitter metabolism in the central nervous system. This is an accepted practice in the assessment of neurotransmitter disorders.^27^ However to obtain these samples would be impractical in patients with AKU owing to their complex musculoskeletal comorbidities and unethical in the absence of clinical evidence of altered cognitive decline or increased severity of depression. Previous studies in patients with AKU have made assessments of neurotransmitter metabolism in urine and reported increased urinary excretion of the dopamine metabolite, 3‐methoxytyramine (3‐MT) in patients receiving nitisinone on a short^3^ and long term^28^ (unpublished data from SONIA‐2 clinical trial) basis. These studies concluded that the marked increase in 3‐MT is likely to reflect altered peripheral catecholamine metabolism. In the long‐term study^28^ Beck Depression Inventory‐II (BDI‐II) scores were also reported as a clinical assessment of mood over a 2‐year period, reassuringly no concurrent change in BDI‐II scores was observed.

Herein for the first time we present Wechsler Adult Intelligence Scale‐IV (WAIS‐IV) data^29^ as an assessment of cognitive functioning, over a 6‐year period, and BDI‐II scores, over a 7‐year period, as an assessment of depression^30^, ^31^ in patients with AKU following treatment with nitisinone. Both assessments are reported with serum tyrosine concentrations over the same period.

## 2 METHODS

### 2.1 Patients included in longitudinal survey

#### 2.1.1 Protocol for patients who attend the National AKU Centre (NAC) for treatment with nitisinone

The protocol for treatment at the NAC has been reported previously.^28^ In brief, this requires that a patient has a confirmed diagnosis of AKU (they must be a resident of England or Scotland, and be over the age of 16 years) in order to be commenced on a 2 mg dose of nitisinone, on alternative days for the first 3 months, and increased to 2 mg daily thereafter. Clinical and biochemical assessments are repeated on an annual basis to monitor response to therapy. Part of these assessments included the evaluation of cognitive functioning and depression using the WAIS‐IV^29^ and the BDI‐II,^30^, ^31^ respectively. These tests were carried out by the Department of Psychology in the University of Liverpool between 2012 and 2019. Not all patients included in this study completed WAIS‐IV and BDI‐II assessments on an annual basis as proposed by the NAC.

### 2.2 WAIS‐IV

The WAIS‐IV is a comprehensive test of cognitive functioning (comprising 10 subtests within four scales), which produces composite scores for (i) verbal comprehension: the Verbal Comprehension Index (VCI), (ii) perceptual reasoning: the Perceptual Reasoning Index (PRI), (iii) working memory: the Working Memory Index (WMI), and (iv) processing speed: the Processing Speed Index (PSI).^29^ From these index scores the Full Scale Intelligence Quotient (FSIQ), the General Ability Index (GAI) or neither may be calculated, depending on the variance between the index scores. Herein FSIQ and GAI are not reported due to the variability in the index scores. The index scores provided by the WAIS‐IV are standardised to a mean of 100, and a standard deviation of 15 with percentile equivalents and confidence intervals. Scores of ≥116 indicate normative strength, of 85–115 normal limits, and of ≤84 normative weakness.

Sixty three patients on nitisinone (27 females, mean age [±standard deviation] 55.7 [13.7] years [range 26–79]; 36 males, mean age 60.3 [9.6] years [range 19–75]) were included in this retrospective longitudinal survey reporting WAIS‐IV subtest scores over a 6‐year period.

### 2.3 BDI‐II

Information on BDI‐II has been previously been reported by our group.^28^ This self‐report instrument includes 21 items; each item has four different statements placed in order of severity from zero to three. Symptoms in the inventory include: Pessimism, Irritability, Past Failure, Punishment Feelings, Self‐Criticalness, Self‐Dislike, Crying, Suicidal Thoughts or Wishes, Changes in Sleep Pattern, Guilty Feelings and Changes in Appetite. Conventional cut‐offs used to categorise patients are: 0–13 (minimal depression), 14–19 (mild depression), 20–28 (moderate depression) and 29–63 (severe depression), with a maximum score of 63.

Seventy four patients (32 females, mean age [±standard deviation] 56.1[13.2] years [range 26–79]; 42 males, mean age 51.5[16.3] years [range 19–70]) were included in this retrospective longitudinal study reporting the BDI‐II data over a 7‐year period.

### 2.4 Biochemical analysis

Serum samples (S‐monovette, Sarstedt, Germany) were collected from patients at each visit to the NAC. Samples were centrifuged (10 min at 3000 rpm) and stored at −20°C until analysis. Samples were collected following an overnight fast (at least 8 h). Patients' dietary intake of protein was managed through a 7‐day food diary by a combination of lower protein in diet and phenylalanine/tyrosine free amino acid supplements. Serum tyrosine was measured using liquid chromatography tandem mass spectrometry as previously reported.^32^ Serum tyrosine was not measured on all patients that had WAIS‐IV and BDI‐II assessments, only where samples were available.

## 3 STATISTICAL ANALYSIS

### 3.1 WAIS‐IV

Statistical analyses were carried out using SAS software (version 9.4, Buckinghamshire, UK) and GraphPad InStat (version 3.10, 2009, CA, USA).

A longitudinal statistical model (linear mixed model) was used to assess changes in WAIS‐IV index scores over the period studied. This assessed change within an individual over time where repeat measures were available (see Reference 33 for a comprehensive review on longitudinal data analysis). This type of model has two stages of analysis that are conceptually distinct, but are combined within a single statistical model. In the first stage, within‐individual change is considered in terms of a summary of the changes in the repeated measurements on each individual during the period of time assessed. The second stage estimates within‐individual change and relates this to inter‐individual differences in selected covariates (e.g., gender, age and treatment group). A *p* value < 0.05 was deemed significant.

Spearmans rank correlation was used to assess if WAIS‐IV sub‐index tests and serum tyrosine concentrations were correlated. A *p* value < 0.05 was deemed significant. Correlation analysis was only performed when both tyrosine and WAIS‐IV sub‐index scores were available.

### 3.2 BDI‐II

Statistical analyses were performed using GraphPad InStat (version 3.10, 2009, CA, USA) and Analyse‐It for Microsoft Excel (version 2.20 Analyse‐it Software, Ltd., Leeds, UK).

Kolmogorov–Smirnov testing was performed to assess if BDI‐II and biochemical data were normally distributed. Wilcoxon matched‐pairs signed‐ranks test (two‐tailed) was used to assess significant differences in BDI‐II scores at baseline (pre‐nitisinone) and at each year following treatment with nitisinone in patients that undertook BDI‐II assessments. A one‐way ANOVA was used to assess significant differences in patients that had BDI‐II scores for all visits from baseline through to 3, 4, 5, 6 and 7 years. Spearmans rank correlation was used to assess if BDI‐II scores and serum tyrosine concentrations were correlated. A *p* value < 0.05 was deemed significant. Correlation analysis was only performed when both tyrosine and BDI‐II scores were available.

### 3.3 Tyrosine

Changes in serum tyrosine concentrations were assessed using a Kruskal–Wallis test with a Dunn's multiple comparisons post hoc test pre‐ and post‐treatment with nitisinone, a *p* value < 0.05 was deemed significant.

## 4 RESULTS

### 4.1 WAIS‐IV

At the time of data collection 63 patients attending the NAC were receiving nitisinone therapy and completed baseline WAIS‐IV assessments (i.e., had all four index scores). Measures were taken at baseline (pre‐nitisinone) and at six subsequent annual visits. No significant differences (*p* > 0.05 in all comparisons) were seen in any aspects of the WAIS‐IV index scores over this period. There were no significant decreases in function on any of the sub‐index scores.

In order to further investigate the WAIS‐IV data, a 6‐year analysis was considered the most appropriate to report in detail as it balanced the two aspect of sample size and validity of the data. A longitudinal model was fitted to each outcome component to see if there was a statistically significant change over time. All four models were fitted using a 6‐year analysis and adjusted for by important characteristic factors (i.e., sex and age at baseline). The results are presented alongside 95% confidence intervals (Tables 1 and 2). If the confidence interval contains ‘0’ it indicates that it is not statistically significant at the 5% level, and therefore there is no difference from baseline.

**TABLE 1:** Results for 6‐year analysis of WAIS‐IV index scores adjusted by age at baseline (pre‐nitisinone) using a longitudinal statistical model (linear mixed model)

Longitudinal model	WAIS‐IV index scores							
	Verbal comprehension		Perceptual reasoning		Working memory		Processing speed	
	Estimate (SE)	95% CI	Estimate (SE)	95% CI	Estimate (SE)	95% CI	Estimate (SE)	95% CI
Intercept (*n* = 43)	92.58 (8.31)	(75.39, 109.77)	111.62 (7.16)	(96.81, 126.43)	100.70 (6.53)	(87.19, 114.21)	107.93 (6.40)	(94.69, 121.16)
Year 2 (*n* = 39)	3.16 (1.22)	(0.64, 5.68)	−0.76 (1.53)	(−3.93, 2.41)	1.40 (1.33)	(−1.35, 4.15)	3.24 (1.16)	(0.84, 5.64)
Year 3 (*n* = 36)	1.96 (1.18)	(−0.48, 4.40)	1.76 (1.41)	(−1.16, 4.68)	3.40 (1.60)	(0.09, 6.71)	1.64 (1.80)	(−2.08, 5.36)
Year 4 (*n* = 29)	3.16 (1.55)	(−0.05, 6.37)	2.00 (1.52)	(−1.14, 5.14)	3.80 (1.16)	(1.40, 6.20)	5.76 (1.79)	(2.06, 9.46)
Year 5 (*n* = 26)	2.88 (1.22)	(0.36, 5.40)	4.20 (1.40)	(1.30, 7.10)	2.12 (1.32)	(−0.60, 4.84)	7.24 (2.06)	(2.97, 11.51)
Year 6 (*n* = 15)	5.84 (1.55)	(2.64, 9.04)	4.92 (1.16)	(2.51, 7.33)	3.20 (1.47)	(0.15, 6.25)	8.64 (1.93)	(4.64, 12.64)
Age	0.26 (0.17)	(−0.10, 0.61)	−0.07 (0.15)	(−0.37, 0.23)	−0.07 (0.13)	(−0.34, 0.20)	−0.14 (0.13)	(−0.41, 0.14)

*Note*: Data were analysed using a longitudinal statistical model. Scores of ≥116 indicate normative strength, 85–115 normal limits and ≤84 normative weakness.

Abbreviations: CI, confidence interval; SE, standard error.

**TABLE 2:** Results for 6‐year analysis of WAIS‐IV index scores adjusted by sex at baseline (pre‐nitisinone) using a longitudinal statistical model (linear mixed model)

Longitudinal model	WAIS‐IV index scores							
	Verbal comprehension		Perceptual reasoning		Working memory		Processing speed	
	Estimate (SE)	95% CI	Estimate (SE)	95% CI	Estimate (SE)	95% CI	Estimate (SE)	95% CI
Intercept (*n* = 43)	100.51 (3.39)	(93.51107.52)	105.81 (3.03)	(99.53, 112.09)	96.03 (2.85)	(90.12, 101.93)	99.95 (2.63)	(94.51, 105.38)
Year 2 (*n* = 39)	3.16 (1.22)	(0.64, 5.68)	−0.76 (1.53)	(−3.93, 2.41)	1.40 (1.33)	(−1.35,4.15)	3.24 (1.16)	(0.84, 5.64)
Year 3 (*n* = 36)	1.96 (1.18)	(−0.48, 4.40)	1.76 (1.41)	(−1.16, 4.68)	3.40 (1.60)	(0.09, 6.71)	1.64 (1.80)	(−2.08, 5.36)
Year 4(*n* = 29)	3.16 (1.55)	(−0.05, 6.37)	2.00 (1.52)	(−1.14, 5.14)	3.80 (1.16)	(1.40, 6.20)	5.76 (1.79)	(2.06, 9.46)
Year 5 (*n* = 26)	2.88 (1.22)	(0.36, 5.40)	4.20 (1.40)	(1.30, 7.10)	2.12 (1.32)	(−0.60, 4.84)	7.24 (2.06)	(2.97, 11.51)
Year 6 (*n* = 15)	5.84 (1.55)	(2.64, 9.04)	4.92 (1.16)	(2.51, 7.33)	3.20 (1.47)	(0.15, 6.25)	8.64 (1.93)	(4.64, 12.64)
Females	13.32 (5.79)*	(1.34, 25.29)*	9.69 (4.84)	(−0.33, 19.70)	5.34 (4.61)	(−4.20, 14.88)	6.62 (4.60)	(−2.89, 16.13)

*Note*: Scores of ≥116 indicate normative strength, 85–115 normal limits, and ≤84 normative weakness.

Abbreviations: CI, confidence interval; SE, standard error.

* *p* ≤ 0.05, *p* < 0.05 deemed significant.

The longitudinal model when adjusted for age and sex at baseline showed that there were no significant differences at a level of 5% across all of the WAIS‐IV index scores over the 6‐year period, except for a significant (*p* < 0.05) improvement in verbal comprehension being observed in females when compared to baseline (Table 2).

Serum tyrosine concentrations were also measured at each visit (Table 3). At baseline tyrosine was within the healthy population reference range^34^ and increased significantly following nitisinone therapy at all annual follow ups (*p* < 0.001, all cases).

**TABLE 3:** Mean (±standard deviation) serum tyrosine concentrations (μmol/L) at baseline (pre‐nitisinone) and following nitisinone treatment (2 mg daily)

Variable	Time points													
	Baseline		1 year		2 years		3 years		4 years		5 years		6 years	
	*n*	Mean (±SD)	*n*	Mean (±SD)	*n*	Mean (±SD)	*n*	Mean (±SD)	*n*	Mean (±SD)	*n*	Mean (±SD)	*n*	Mean (±SD)
[Tyrosine]^a^	63	60.5 (65.2)	43	607.3 (262.4)*	39	617.1 (290.4)*	36	692.8 (279.8)*	29	759.0 (243.6)*	26	713.7 (110.7)*	15	666.5 (183.5)*

Abbreviation: SD, standard deviation.

^a^ Reference range 29–92 μmol/L^34^

* *p* < 0.001 when time point compared to baseline. Serum tyrosine concentrations are only included where WAIS‐IV sub‐index scores are also available.

Table 4 provides a summary of correlations performed between serum tyrosine and WAIS‐IV index scores. Over the 6‐year period WAIS‐IV index scores and serum tyrosine were not correlated pre‐ or post‐nitisinone treatment.

**TABLE 4:** WAIS‐IV index scores correlated with serum tyrosine concentration (μmol/L) over 6 years

Variable	Time points						
	Baseline	1 year	2 years	3 years	4 years	5 years	6 years
	rs	rs	rs	rs	rs	rs	rs
VCI vs. [Tyrosine]	−0.081	−0.1	0.04	0.03	0.03	0.09	0.33
PRI vs. [Tyrosine]	−0.19	−0.09	−0.17	−0.08	−0.20	−0.15	−0.04
WMI vs. [Tyrosine]	0.09	−0.02	−0.10	0.05	−0.39	0.15	−0.18
PSI vs. [Tyrosine]	0.03	0.08	−0.11	0.09	−0.22	−0.11	−0.22

Abbreviations: PSI, Processing Speed Index; PRI, Perceptual Reasoning Index; VCI, Verbal Comprehension Index; WMI, Working Memory Index.

### 4.2 BDI‐II

**FIGURE 1:**
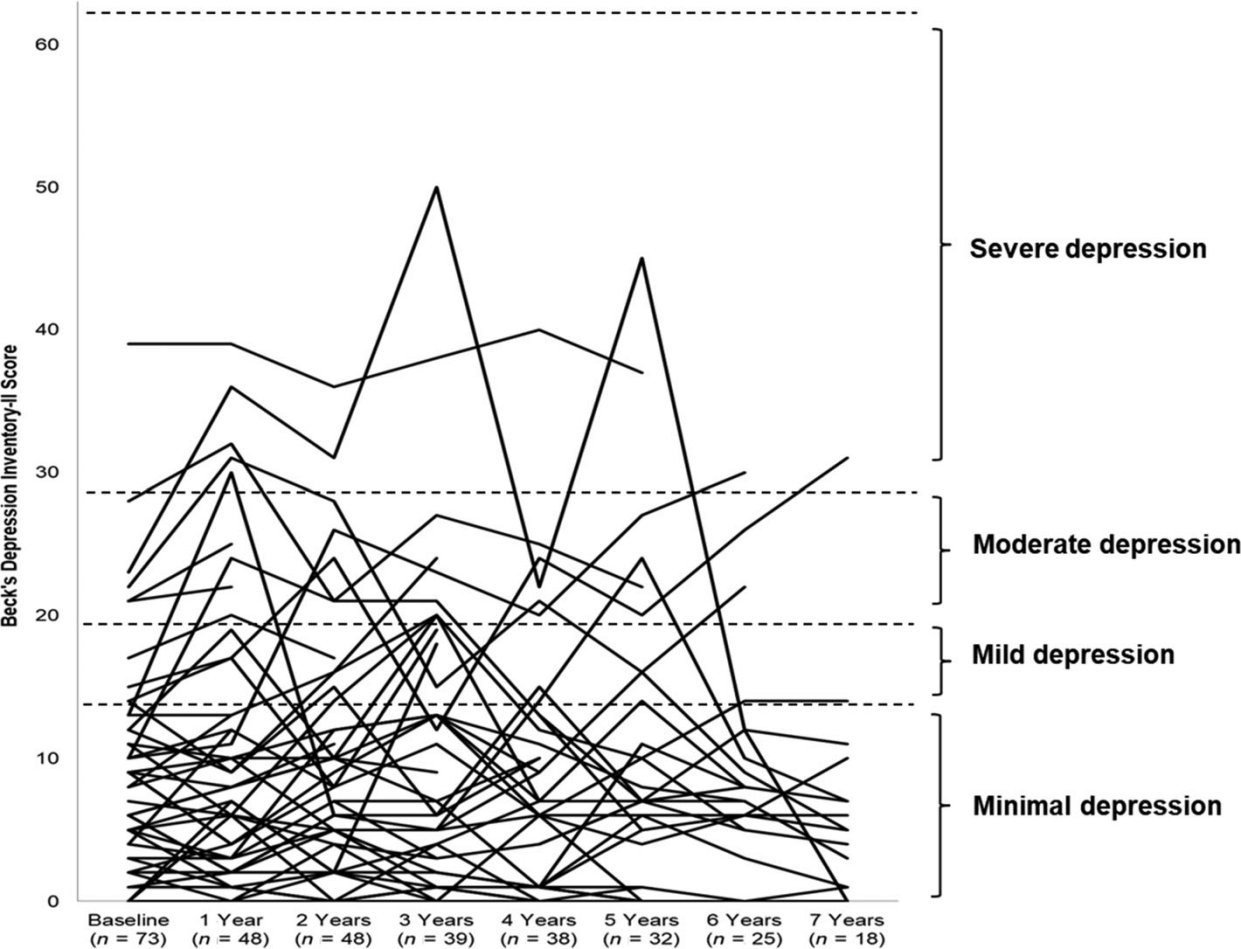
Summary of BDI‐II scores over 7‐year period. No significant differences were observed when baseline and subsequent visits were compared. BDI‐II scoring: minimal depression (0–13 points); mild depression (14–19 points); moderate depression (20–28 points); severe depression (29–63 points). See Table S2 for *p* values

Data from 74 patients are reported herein, at baseline (pre‐nitisinone) assessments were carried out in 73 patients. At each annual follow up not all patients had BDI‐II assessments over the 7‐year period studied. Those patients that had assessments are shown in Figure 1 and Table 5. There were no significant differences in mean BDI‐II scores when baseline scores were compared to annual mean BDI‐II scores over the 7‐year period (*p* > 0.05 in all cases) (i.e., post nitisinone). At 3, 5 and 7 years there were 35, 24, 10 patients that had all annual BDI‐II assessments, in all cases mean BDI‐II scores were also not significantly different (*p* > 0.05 in all cases, see Table S1).

**TABLE 5:** Mean (±standard deviation) BDI‐II scores and tyrosine concentrations (μmol/L) at baseline (pre‐nitisinone) and at 1–7 years following nitisinone treatment (2 mg daily)

Variable	Time points							
	Baseline	1 year	2 years	3 years	4 years	5 years	6 years	7 years
	(*n* = 73)^a^	(*n* = 48)	(*n* = 47)	(*n* = 38)	(*n* = 34)	(*n* = 32)	(*n* = 24)	(*n* = 12)
[BDI‐II]^b^	10.2 (9.4)	10.7 (10.3)	9.5 (8.7)	11.3 (11.1)	9.1 (8.7)	10.7 (10.7)	8.8 (7.6)	7.1 (7.9)
[Tyrosine]^c^	50.3 (14.2)	607.9 (269.3)*	638.3 (310.4)*	698.4 (296.4)*	732.9 (259.6)*	745.1 (184.3)*	688.1 (255.9)*	709.2 (179.6)*
BDI‐II vs. [Tyrosine] (rs)	0.19	−0.068	0.16	−0.22	0.00053	0.10	0.19	0.13

*Note*: Serum tyrosine concentrations are only included in this table, where BDI‐II scores are also available; the number of patients included in Figure 1 are different to this as some of these patients did not have tyrosine measured.

^a^ Two patients excluded as already on nitisinone when baseline sample collected.

^b^ BDI‐II scoring: minimal depression (0–13 points); mild depression (14–19 points); moderate depression (20–28 points); severe depression (29–63 points).

^c^ Reference range 29–92 μmol/L.^34^

* *p* < 0.001 when time point compared to baseline.

Eight patients (four males, four females) were included at baseline in the statistical analysis, but were removed from subsequent analysis as they did not receive nitisinone treatment (seven patients were from Wales and one from Ireland – these patients are not eligible for nitisinone treatment, but attended the NAC for clinical and biochemical assessment). These patients serve as a small control group (Table S3), none of the patients had all annual BDI‐II assessments. Statistical analysis was not performed in this subgroup as the number of patients was small.

42/74 of the patients in this longitudinal were on pain relief (56.7%): 18 patients were on 1 agent; 12 patients were on 2 agents; 9 patients were on 3 agents and 3 patients were on 4 agents. Additionally 8/74 (10.8%) patients were on anti‐depressants at baseline. 5/8 received nitisinone and remained on anti‐depressants throughout the study. The other three patients did not receive nitisinone (Welsh [*n* = 2] and Irish [*n* = 1]).

At baseline 72% of patients were categorised as having minimal depression (Table 6), this increased over the 7‐year period studied to 83.3%. The number of patients in the mild depression and moderate depression categories decreased over the same period. There were a small number of patients in the severe category at all‐time points, which decreased over the period studied, but so too did the overall number of patients at each time point.

**TABLE 6:** Categorisation of BDI‐II scores at baseline (pre‐nitisinone) and 1–7 years following 2 mg nitisinone daily

BDI‐II depression category	Time points															
	Baseline		1 year		2 years		3 years		4 years		5 years		6 years		7 years	
	*n*	%	*n*	%	*n*	%	*n*	%	*n*	%	*n*	%	*n*	%	*n*	%
Minimal	53	72.6	36	75.0	36	75.0	27	69.2	30	78.9	23	71.9	21	84.0	15	83.3
Mild	8	11.0	3	6.3	5	10.4	3	7.7	2	5.3	3	9.4	1	4.0	2	11.1
Moderate	9	12.3	4	8.3	5	10.4	7	17.9	5	13.2	4	12.5	2	8.0	0	0.0
Severe	3	4.1	5	10.4	2	4.2	2	5.1	1	2.6	2	6.3	1	4.0	1	5.6

Serum tyrosine concentrations were in the normal reference range^34^ in all of those patients not receiving nitisinone at baseline (two patients were excluded from the data as they were on nitisinone at the time baseline samples were collected). Following treatment with nitisinone tyrosine concentrations increased significantly at all visits over the 7‐year period studied (*p* < 0.001, all cases). BDI‐II scores and serum tyrosine were correlated at baseline and over the 7‐year period studied. In all cases no correlation was observed (Table 5).

## 5 DISCUSSION

Whilst there is debate about the impact that nitisinone‐induced hypertyrosinaemia may have on intellectual functioning in HT‐1,^11^, ^17^, ^18^, ^19^, ^20^ nothing has been reported on AKU despite the presence of supraphysiological tyrosine concentrations following treatment with nitisinone.

This highly unique and original retrospective longitudinal survey is the first to report WAIS‐IV index scores^29^ in patients with AKU following treatment with nitisinone. Additionally, BDI‐II scores, as a measure of depression severity,^30^, ^31^ are also presented over a much longer period than previously reported.^28^

### 5.1 WAIS‐IV

The use of WAIS‐IV is a widely accepted tool in clinical practice to assess intellectual functioning. The data presented herein demonstrates that nitisinone‐induced hypertyrosinaemia did not alter PRI, WMI or PSI over a 6‐year period, although a significant difference was observed for VCI after adjustment for sex. These findings are highly reassuring and provide confidence to patients and clinicians alike about the safe use of nitisinone. A limitation in the WAIS‐IV data reported in this study is that FSIQ and GAI could not always be calculated due to the variability in index scores.

Nonetheless these findings are still important, and in keeping with a recent report on HT‐1^11^ where a low incidence of cognitive or development impairment was observed over a 15‐year period in patients with HT‐1 treated with nitisinone (nine out of 315 patients showed cognitive or development impairment; seven out of nine of these patients were treated with nitisinone by 6 months of age). However a limitation of this study is that assessment of cognitive function is not always standardised. In contrast to this in a smaller study^21^ it was demonstrated that patients with HT‐1 had several behaviour problems and a lower health related quality of life.

Like any clinical investigation one must consider limitations. In using the WAIS‐IV assessments there is the potential for ‘practice effects’—that is a change in test performance which results from repeated administration (i.e., annual assessment) of the cognitive tests. It has been reported that a patient's neuropsychological performance on an assessment can improve due to the re‐administration of the same testing instrument.^35^, ^36^ Normative gains on individual sub‐tests are likely to be due to the effect of practice and not to real gains in intellectual functioning.^37^ Additionally this is further complicated by the fact that patients with AKU are individuals in their own right and so there is considerable variability in their cognitive functioning. Another important factor that may have influenced WAIS‐IV assessments is that patients with AKU are known to have chronic pain (56.7% of patients are on pain relief medications in this study), which is known to alter cognitive outcomes,^38^ emerging evidence has also shown the multi‐dimensional effects of pain on various cognitive domains.^39^

### 5.2 BDI‐II

BDI‐II scores did not significantly increase over the 7‐years studied when compared to baseline in men and women, and there were no gender differences. This was in the mean data and those patients that were followed over 3, 5 and 7 years. This is in keeping with previous findings,^28^ but in this study we report over a much longer period in a larger cohort of patients providing further support for the safe use of nitisinone in patients with AKU. The data presented provide additional evidence that nitisinone‐induced hypertyrosinaemia does not lead to worsening depression.

### 5.3 Hypertyrosinaemia

As expected significant hypertyrosinaemia was observed, which is in keeping with previous reports following treatment with nitisinone.^1^, ^3^, ^4^, ^5^, ^6^, ^7^, ^8^, ^9^

An important finding in relation to this is that none of the WAIS‐IV index scores or BDI‐II scores were correlated with serum tyrosine. The lack of a correlation between WAIS‐IV index scores and tyrosine supports the findings of a study in patients with HT‐1 where no correlation was observed between serum tyrosine concentration and intelligence quotient in patients receiving nitisinone therapy.^22^

The absence of a correlation between BDI‐II scores and tyrosine is in agreement with a previous study reporting over a 2‐year period in patients attending the NAC.^28^ The absence of a correlation between the two scales and tyrosine suggests that tyrosine is not associated with a decline in cognitive functioning or an increase in severity of depression.

## 6 CONCLUSIONS

The findings of this retrospective longitudinal survey suggest that treatment with nitisinone and the consequent hypertyrosinaemia does not affect cognitive functioning or severity of depression in AKU patients.

## CONFLICT OF INTERESTS

The authors have no conflict of interest.

## ETHICS STATEMENT

Data collection and analyses at the NAC was approved by Liverpool University Hospitals NHS Foundation Trust Audit Committee (Audit no. ACO3836). As these data were collected as part of the clinical service ethical approval was not required from the Health Regulatory Authority and North West—Haydock Research Ethics committee (United Kingdom). Patients were informed verbally and through patient information leaflets about the activities of the NAC, including that data may be used for publication and within the patient information leaflet used at the NAC.

All procedures reported in this manuscript were in accordance with the Code of Ethics of the World Medical Association and the Helsinki Declaration of 1975, as revised in 2000.

## Supporting information


**Data S1 Table S1**: Summary of Wilcoxon matched paired *t*‐tests comparing baseline BDI‐II scores to BDI‐II scores from follow‐up visits. *p* < 0.05 deemed significant.
**Table S2.** Summary of one‐way ANOVA comparing baseline BDI‐II scores to BDI‐II scores from follow‐up visits in patients who had scores from all visits. *p* < 0.05 deemed significant.
**Table S3.** BDI‐II scores in patients who did not receive nitisinone therapy. Statistical analysis not performed as groups are too small.

## Data Availability

My manuscript has no associated data.
