# Supplementary material for: Long‐term low dose nitisinone therapy in adults with alkaptonuria shows no cognitive decline or increased severity of depression
Source: JIMD Rep. 2022 Mar 17;63(3):221–30. doi: 10.1002/jmd2.12272 (PMC8995840; doi:10.1002/jmd2.12272)
Supplement: Supplementary file 1 — Data S1 Table S1: Summary of Wilcoxon matched paired t‐tests comparing baseline BDI‐II scores to BDI‐II scores from follow‐up visits. p < 0.05 deemed significant. Table S2. Summary of one‐way ANOVA comparing baseline BDI‐II scores to BDI‐II scores from follow‐up visits in patients who had scores from all visits. p < 0.05 deemed significant. Table S3. BDI‐II scores in patients who did not receive nitisinone therapy. Statistical analysis not performed as groups are too small. [file JMD2-63-221-s001.docx]

**Supplementary material**

| **Comparison to baseline** | **Males** | | **Females** | |
| --- | --- | --- | --- | --- |
|  | **n** | **p** | **n** | **p** |
| 1-Year | 28 | 0.27 | 20 | 0.11 |
| 2-Years | 28 | 0.32 | 20 | 0.26 |
| 3-Years | 24 | 0.54 | 15 | 0.24 |
| 4-Years | 26 | 0.51 | 12 | 0.76 |
| 5-Years | 19 | 0.44 | 13 | 0.26 |
| 6-Years | 16 | 0.70 | 9 | 0.06 |
| 7-Years | 11 | 0.82 | 7 | 0.81 |

**Table S1.** Summary of Wilcoxon matched paired t-tests comparing baseline BDI-II scores to BDI-II scores from follow-up visits. p<0.05 deemed significant.

| **Patients with BDI-II scores from all visits at:** | **n** | **p** |
| --- | --- | --- |
| 3-Years | 35 | 0.68 |
| 4-Years | 28 | 0.91 |
| 5-Years | 24 | 0.94 |
| 6-Years | 10 | 0.99 |

**Table S2.** Summary of 1-way ANOVA comparing baseline BDI-II scores to BDI-II scores from follow-up visits in patients who had scores from all visits. p<0.05 deemed significant.

| **Visit** | **n** | **Mean** | **SD** |
| --- | --- | --- | --- |
| Baseline | 8 | 11.8 | 7.1 |
| 1-Year | 5 | 12.6 | 5.3 |
| 2-Years | 5 | 10.8 | 7.4 |
| 3-Years | 5 | 10.6 | 5.6 |
| 4-Years | 4 | 12.3 | 8.0 |
| 5-Years | 2 | 15.0 | 7.1 |
| 6-Years | 2 | 15.5 | 14.8 |
| 7-Years | 2 | 17.5 | 19.1 |

**Table S3.** BDI-II scores in patients who did not receive nitisinone therapy. Statistical analysis not performed as groups are too small.
